# Mechanical Properties of Polylactide Admixed with Carbon Nanotubes or Graphene Nanopowder

**DOI:** 10.3390/ma14205955

**Published:** 2021-10-09

**Authors:** Piotr Szatkowski, Leszek Czechowski, Jacek Gralewski, Martyna Szatkowska

**Affiliations:** 1 Faculty of Materials Science and Ceramics, AGH University of Science and Technology, 30-059 Krakow, Poland; pszatko@agh.edu.pl (P.S.); szatkowska.martyna1997@gmail.com (M.S.); 2 Department of Strength of Materials, Lodz University of Technology, 90-924 Lodz, Poland; 3 Institute of Social Sciences and Management of Technologies, Lodz University of Technology, 90-924 Lodz, Poland; jacek.gralewski@p.lodz.pl

**Keywords:** carbon nanotubes, grapheme nanopowder, mechanical properties, polylactic acid

## Abstract

The aim of this work was to verify the material properties of polylactic acid (PLA) with an addition of carbon nanotubes (CNTs) or graphene nanopowder (GNP). The pure polylactide and admixed polylactide samples were subjected to chemical–physical tests to determine their stiffness and strength parameters. The tensile and impact tests were performed on samples without UV (ultraviolet) treatment and after UV treatment, in a physiological saline solution. The investigations were composed of two stages. The first one was related to the examination of the properties of pure polylactide, denoted as the following: 3001D, 4032D, and 4043D. The second stage was based on an analysis of the properties of PLA 4032D with an admixture of GNP or CNTs, at 0.1 wt.% and 0.5 wt.%. By comparing the strength and the stiffness of pure samples with samples with the considered admixtures, an essential increase was not observed. However, it is stated that the presence of GNP and CNTs in the samples positively influenced the resistance of the materials to the ageing process.

## 1. Introduction

The contemporary industry of modern materials is developed with huge tempo everywhere. At present, polymer materials are still used in many branches, though they are weakly biodegradable. The essential types of materials are composite materials, which are produced from basic materials, such as polymers and modifiers, enhancing general mechanical properties or enabling an adoption of polymers to the appropriate field. The alternative of conventional polymer materials is biopolymers that are characterized by similar mechanical properties to conventional polymers, but they have fundamental assets, namely, they naturally decompose in a short amount of time. Among biodegradable biopolymers, a well-known biopolymer is polylactic acid (PLA), which is featured by good thermo-plasticity and moderately high mechanical properties. Bio-composites are regarded as polymer composites that are made of plants. There is also a possibility to produce biodegradable biopolymers from bacteria, mushrooms, or crustacean shells. Composite materials are usually made up of a matrix and reinforcement. The reinforcement constitutes a material that has better mechanical properties to ensure appropriate strength of the composite material. A unique material with extremely good mechanical properties is graphene, which distinguishes itself with extraordinary thermal and electric conductivity. The main components of biocomposites are fibers and bio-fibers made of glass, graphite, and carbon or organic fibers.

Figueroa-Velarde et al., in [1], analyzed the mechanical properties of PLA samples filled with agave fibers. The samples were produced by fused deposition modeling (FDM)-based 3D printing. They considered the differential scanning calorimetry, morphology, water uptake, density variation and composting of the biocomposites. The proliferation of 3D printing helped PLA become the lead polymer used in this technique. Previously, PLA was used very occasionally, mainly in medicine, for use as surgical sutures. Prajapati et al., in [2], investigated fiber-reinforced polymer composites (FRPC), in order to verify the influence of the volume of fiber fractions on their impact strength. Due to its high crystallinity, PLA is a brittle material. This is one of the most important disadvantages of this polymer in terms of its various potential uses (e.g., as packaging and food containers). The impact resistance is improved by modifying PLA with natural fibers or plasticizers. However, the improvement in this property is not always at a satisfactory level [3,4,5,6]. Research suggested that the brittleness of PLA is due to the low entanglement density (Ve) and the high characteristic ratio (C∞), which is a measure of chain stiffness [7,8]. Furthermore, it has been reported that the entanglement density of polymer chains changes during the physical aging process, which will induce a variation in the mechanical properties, especially the toughness and fracture performance, of polymers. Therefore, it is considered that the mechanical properties of PLA are also dependent on the ageing process. Thus, the study on the relationship between physical ageing, morphology, and mechanical properties can probably provide some approaches to improve the toughness of PLA-based materials [9,10]. The authors in [11,12] presented a review of the work relating to the usage of natural fibers to strengthen polymer composite materials. It was confirmed that natural fibers are lighter than synthetic ones. These review articles reveal the chemical, mechanical and physical properties of plant fibers, and the techniques of polymer reinforcement. The use of natural fibers in the case of PLA is the best choice (due to the complete biodegradability of the composite); however, natural fibers have their drawbacks (lower mechanical parameters and the spread of properties depending on the harvesting conditions of fiber-forming plants). Polylactide degrades over time; the literature shows that various modifiers can affect the duration of biodegradation, and that they can be used to regulate and plan the decomposition time of the material [3,13]. One of these degradation time regulators may be carbon nanoforms [14]. Sanes et al., in [15], studied the nanocomposite materials containing a thermoplastic matrix with different forms of graphene or graphene oxide nanofillers. Their results indicate that great interest should be given to biodegradable matrices, such as PLA, and graphene oxide or reduced graphene oxide. Dou et al., in [16], examined the tensile mechanical properties of carbon fiber-reinforced PLA samples manufactured by three-dimensional printing. The results showed that the relative fiber content has a significant influence on the mechanical properties and the ratio of carbon fibers in composites. The authors of paper [17] dealt with the adaptation and improvement of the three-dimensional printer, and the characterization of printed specimens based on carbon and flax fibers. Paper [18] concerns the analysis of a biocompatible graft copolymer (3,4-ethylenedioxythiophene) manufactured by a 3D-printing melt extrusion method. It was revealed that the producer copolymer is characterized by excellent cell growth and maturation of neonatal cardiac myocytes. There are many studies on PLA composites modified with carbon nanoforms [19,20]. However, there are no studies that would check how nanocarbon modifiers affect the mechanical properties of PLA composites/carbon nanoforms during the aging processes, i.e., in the simulation of work and exposure to weather conditions (UV, moisture, microorganisms).

The present work aimed to investigate biodegradable PLA samples admixed with CNTs and GNP. One-directional tensile tests and impact tests were carried out on the samples to determine their mechanical properties. Moreover, the admixed samples were tested after a UV process and UV process in physiological saline solution, to assess the mechanical parameters after an accelerated ageing process. All the tensile curves have been presented, and the characteristic parameters have been sorted into adequate tables. The results of the present work can be profitable in the case of an application of CNTs and GNP in PLA, in order to control the material properties due to the ageing effect. 

## 2. Preparation of Samples

### 2.1. Basic Material

The fundamental material applied for tests was polylactic acid (PLA). This polylactide, produced from polylactic acid or lactide, is a biodegradable, thermoplastic material with mechanical properties close to polystyrene (PS). The synthesis of polyamide from polylactic acid occurs due to direct or azeotropic condensation. The present PLA was prepared by using simple plant sugars, through the process of photosynthesis. Three types of PLA were tested. Series 3 was chosen because it is designated to an injection and it does not contain modifiers. It also has a high MFI. Series 4 is usually designated to foil and has a different composition of PLA chains. They are more irregular and longer than series 3 PLA. Due to the large problem with the film-forming properties of PLA, it was decided that two types of PLA would be tested (unmodified 4032D and with a small amount of 4043D plasticizer).

### 2.2. Applied Admixtures

**Figure 1:**
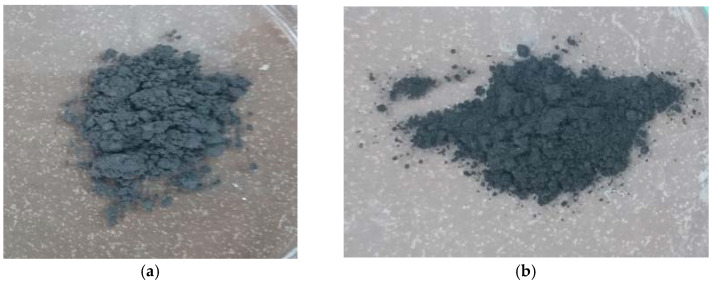
Graphene nanopowder (**a**) and carbon nanotubes (**b**) used as admixtures in PLA samples.

Graphene nanopowder (GNP), as used in the research, consists of graphene flakes with a size of several microns. The producer of this material is the American company Supermarket Graphene. The carbon nanotubes (CNTs) used as a modifier in the polymer are multi-wall carbon nanotubes delivered by Nanostructured & Amorphous Materials (NanoAmor). The technical data provided by the manufacturer indicate that these are industrial nanotubes. Their outer diameters range from 10 to 30 nm, and their lengths are 1–2 µm. Their purity is above 95% (Figure 1).

### 2.3. Samples Manufacturing Process

**Figure 2:**
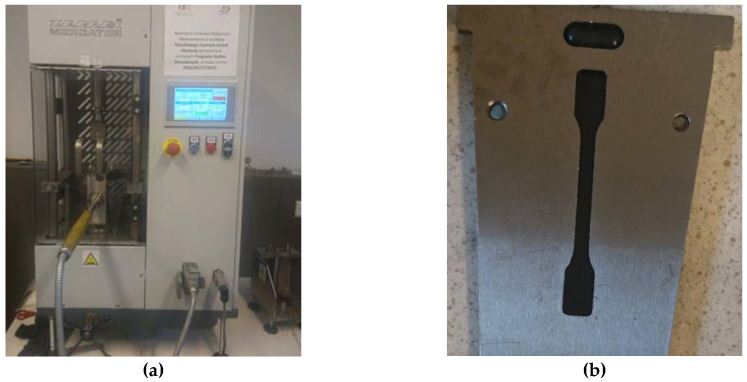
ZAMAK injection molding machine used to producing samples (**a**) and molding die (**b**).

**Figure 3:**
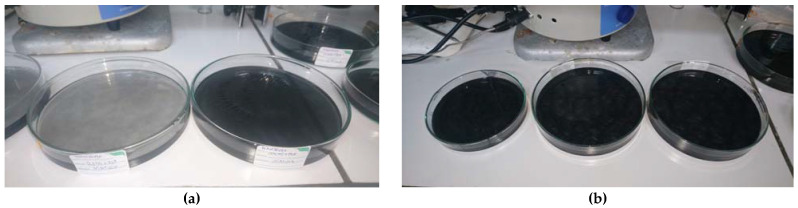
Suspension of PLA and CNTs (**a**) and suspension of PLA and GNP (**b**).

The samples were produced by using an injection molding machine manufactured by the ZAMAK Mercator (Figure 2a), by means of the mold (Figure 2b). The applied machine is a vertical piston injection molding machine. The parameters of the injection molding were set to a manual operating range, appropriately matched to the type of polymer. The injection force and injection time were 12,000 N and 10 s, respectively. The parallel work conducted within this study was to create a polymer composite with a nanocarbon modifier. For this purpose, the solvent method was used. It consisted of dissolving polylactide granules with additives, such as graphene and nanotubes. Fifty grams of PLA, 300 mL of DCM (dichloromethane) solvent, and, depending on the percentage of modifier, the following were prepared: 0.1%–0.05 g graphene/nanotubes and 0.5%–0.025 g graphene/nanotubes. The prepared substance was left for about 24 h until it was completely dissolved (suspensions are shown in Figure 3a,b). The next step in the preparation of this composite was the evaporation of the solvent from the suspension. For this purpose, the Petri dishes filled with the slurry were placed on a preheater and stirred from time to time, until most of solvent was evaporated. After the initial evaporation of the solvent, the round plates were placed in an oven, with the aim of drying the material.

**Figure 4:**
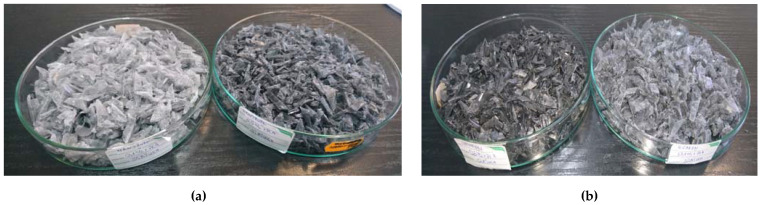
Composite PLA with CNTs in the form of cut pieces (**a**) and composite PLA with GNP in the form of cut pieces (**b**).

**Figure 5:**
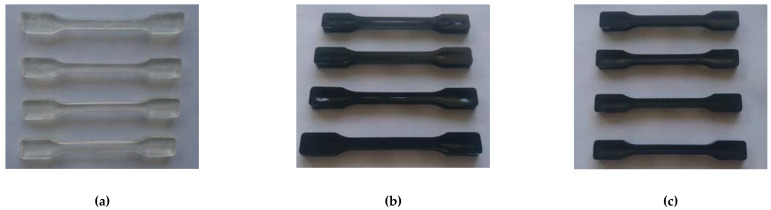
Samples made of pure PLA 4032D (**a**), PLA with the addition of 0.1% and 0.5% of CNTs (**b**) and PLA with the addition of GNP (**c**).

When the materials were suitably dry, they were cut into small pieces (Figure 4) and placed back into the vacuum oven. When the cut composite was sufficiently dry, specimens were manufactured in an appropriate form (Figure 5).

### 2.4. Tensile Test

**Figure 6:**
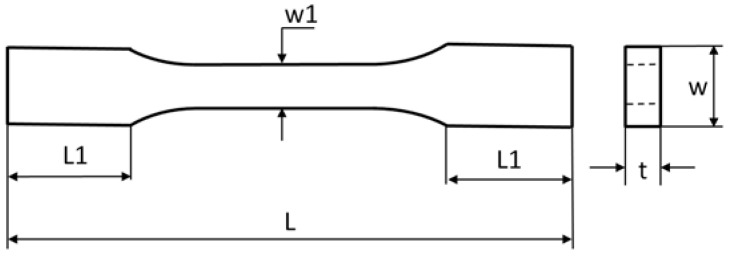
Dimensions of sample subjected to tests.

The tensile tests were carried out using a Zwick 1435 testing machine. This test consisted of axial stretching of the samples with a constant speed of 2 mm/min, until they were completely broken. As a result of stretching the samples, diagrams of force vs. deformation were obtained. The standardized dimensions of the samples are as follows: L = 80 mm, L1 = 15 mm, w1 = 5 mm, w = 10 mm, and mean thickness t = 4.4 mm (Figure 6).

### 2.5. Impact Test

Impact tests are performed in order to determine the ability of materials to bear sudden shock loads. This test is a dynamic test, which enables the characterization of material properties that cannot be detected by using static methods. In this work, a bending impact test was carried out using Charpy’s hammer. The test consisted of breaking the sample supported on supports by one hit of a pendulum hammer. The samples used for the impact tests had the shape of rectangular beams with a rectangular cross-section area. 

### 2.6. Life Durability

**Figure 7:**
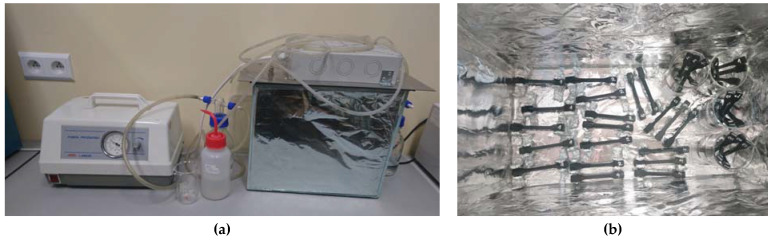
Aging chamber with UV lamps and vacuum pump (**a**) and the view of arranged samples in chamber (**b**).

One of the methods of determining the life of the material is treating the samples with UV rays, allowing the ageing of the material to be determined. The tested samples were placed on appropriate stands in a glass (Figure 7a), a rectangular container, specially lined with reflective foil, as is presented in Figure 7b. The container was closed with a lid, on which one ultraviolet lamp, with a power of 35 W/cm^2^, was placed. In comparison, the average power of UV radiation reaching the Earth from the sun is 62 W/m^2^. This means that one hour of ageing of the material in this special container is equal to 5645 h of exposure to solar radiation. The prepared container was connected to a vacuum pump, which forced the air flow. As a result, the ozone formed in the chamber was pumped out. The samples were irradiated in two ways. The first method was based on loose arrangements of samples on special bases, while the second method involved immersing half the samples in a physiological saline solution (PSS). The entire irradiation process lasted 4 h, while, in the case of samples immersed after 2 h, they were turned. A four-hour exposure is equivalent to approximately one year of exposure to solar radiation.

## 3. Results

### 3.1. Tensile Test

**Figure 8:**
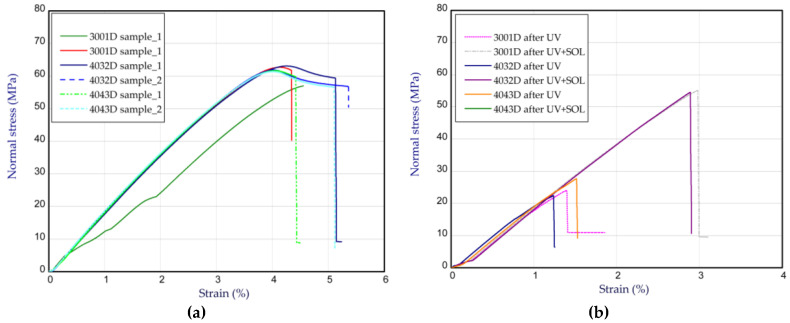
The curves of tension for samples 3001D, 4032D and 4043D before and after UV treatment. (**a**) pure samples, (**b**) single samples.

This subsection shows the tension results for pure PLA, denoted as 3001D, 4032D, and 4043D. The tests were performed for pure samples of different builds (Figure 8a) and for single samples after UV treatment (denotation UV in legend) and UV treatment in solution (denotation UV + SOL in legend). The latter ones are illustrated in Figure 8b. Figure 8 displays the curves of normal stress vs. strain. In general, the maximum stress of all the tested samples (before UV treatment) reached slightly above 60 MPa. In this case, the Young’s moduli were usually registered as close to 1.9 GPa (see Table 1). Based on the results for the samples after UV treatment, a significant drop in the maximum stresses for the considered samples was observed (almost 2.5 times); however, the Young’s moduli remained at the same level. Of course, these data are accomplished for single samples, but the trend was noticed for all the considered types of PLA (3001D, 4032D, and 4043D).

**Figure 9:**
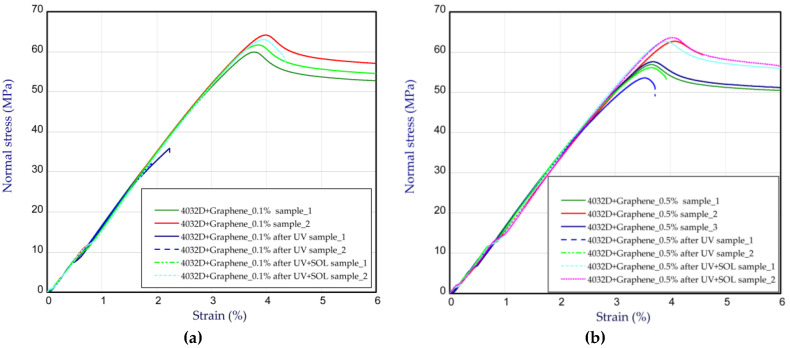
The curves of tensile tests for samples 4032D admixed with graphene at 0.1 wt.% (**a**) and 0.5 wt.% (**b**).

**Figure 10:**
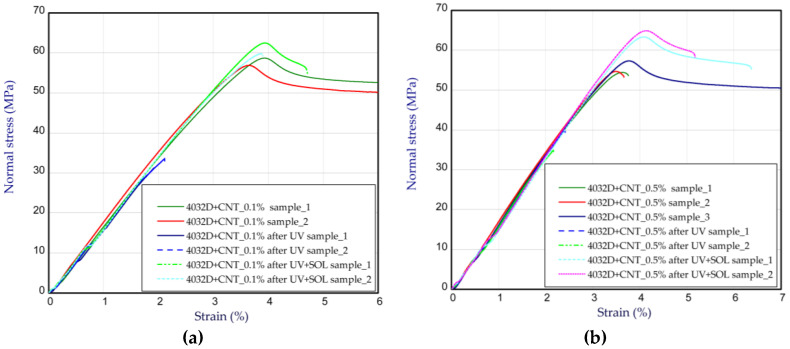
The curves of tensile tests for samples 4032D admixed with CNTs at 0.1 wt.% (**a**) and 0.5 wt.% (**b**).

Figure 9a,b present the tensile characteristics for sample 4032D admixed with GNP. In the cases of 0.1% (Figure 9a) and 0.5% (Figure 9b) of graphene in the samples, a growth in strength is not visible (above 60 MPa, as for pure PLA 4032D). The Young’s modulus is also comparable to pure polylactide. However, with an increase in graphene in the samples, the strength of the material after UV radiation rises in comparison to the strength of the pure samples (for 0.1 wt.% and 0.5 wt.%, there were noticeable increases by 20–30% and 100–120%, respectively). It turned out that a small content of graphene can protect polylactide from the action of UV, i.e., from the ageing process. By taking into consideration the samples after UV treatment in solution, the ultimate stresses are close to the ultimate stresses for the samples without UV treatment. A slight growth in Young’s modulus was observed in the samples after UV treatment (about 5–10%). These characteristic parameters are shown in Table 2. The curves obtained for the samples with CNTs are illustrated in Figure 10. The presence of CNTs in PLA causes a similar effect to the presence of graphene contents. This means that the maximum stresses are greater after UV treatment compared to the maximum stresses of pure PLA samples after UV action. The parameters for the samples with CNTs are given in Table 3. The strains at maximum load usually amounted to about 4% for pure and admixed samples without the UV process, or 1.5% for pure samples subjected to the UV process (see Table 1, Table 2 and Table 3).

### 3.2. Impact Test

The results of the impact tests are given in Table 4, Table 5 and Table 6. Table 4 presents the mean energies and the impact strength (energy referred to the cross-section area of samples in Joul unit) for samples 3001D, 4032D, and 4043D. The highest value of energy needed for dynamic breaking was registered for the 4032D sample. The same values for all the considered samples were obtained for the UV process (0.5 Joul). However, for the samples after UV treatment in solution, the 4032D samples seem to be the most resistant (in this case, 3.25 J). In general, the results of the impact test on the pure samples show that the studied materials after the UV process are characterized by weak resistance to impact. The situation seems to be better for the samples after UV radiation in solution, but the obtained impact strength values are still lower than in the case of the impact strength of samples without UV. Moreover, the conducted tests revealed the level of decrease in the impact strength on samples without nanofillers.

The impact energies for sample 4032D with graphene are shown in Table 5. The content of 0.1% and 0.5% GNP causes an increase in energy, by 19% (6.55 J) and 55% (8.55 J), respectively. Slight growth is also noticeable for samples after the UV process, but for samples after UV treatment in solution, the absorbed energies are close to the energies attained for the admixed samples without UV. A smaller increase in the absorbed energies is observed for samples with CNTs (for 0.1% CNTs—7% and for 0.5% CNTs—29%). Based on the results for samples after the UV process in solution, the obtained energies are minor compared to the impact energies of the samples with graphene. Both the samples with graphene and samples with CNTs indicate similar impact strengths after the UV process.

## 4. Conclusions and Final Remarks

This paper concerns the study of the mechanical parameters of the PLA samples 3001D, 4032D, and 4043D after a UV process. For tests, 4032D samples admixed with GNP and CNTs were examined as well. The admixtures in the samples amounted to 0.1% or 0.5% of modifier. Based on the results, the following can be stated:

- The Young’s moduli among samples 3001D, 4032D and 4043D are comparable. After UV treatment, a slight increase in stiffness, for all the samples, was observed.
- In general, a content of graphene and CNTs of 0.1 wt.% or 0.5 wt.% in samples does not increase their stiffness or strength. Usually, these values are close to the values of pure PLA samples. Nevertheless, the influence of graphene and CNTs in samples is remarkable in tests just after UV treatment. This means that the samples subjected to the UV process had a strength that was 2–3 times less than the samples with UV treatment. However, the additives of GNP or CNTs in the samples enabled a strength close to the strength of the samples without the ageing process to be attained.
- Among the considered PLA samples, 3001D, 4032D, and 4043D, the Young’s modulus and the strength are the most comparable, but the PLA 4032D sample seemed to be slightly stronger.
- The content of graphene in the 4032D polylactide samples allowed a greater impact strength to obtained during the tests.

Polylactide is a material that is being developed very dynamically. There are huge hopes for it, especially in the context of PLA replacing conventional polymers used for packaging and films. Additions of carbon nanoforms make it possible to control their degradation time, so that the material decomposes immediately after its use, and this prevents, as in the case of conventional polymers (PE, PP, PVC), the material remaining in landfills for hundreds of years.

## Figures and Tables

**Table 1:** Characteristic parameters obtained in tensile tests for samples 3001D, 4032D and 4043D.

Parameter	3001D		3001D after UV	3001D after UV + SOL	4032D		4032D after UV	4032D after UV + SOL	4043D		4043D after UV	4043D after UV + SOL
	Sample_1	Sample_2			Sample_1	Sample_2			Sample_1	Sample_2		
Young modulus E [GPa]	1.61	1.87	1.86	2.26	1.88	1.92	2.16	2.14	1.94	1.93	2.15	2.11
Maximum stress R_m_ [MPa]	57.1	62.7	24.1	55.1	63.1	61.8	22.4	54.6	61.7	61.4	27.5	60.6
Strain at maximum load [%]	4.61	4.09	1.39	2.97	4.21	3.97	1.23	2.88	4.04	3.98	1.50	3.93

**Table 2:** Characteristic parameters obtained in tensile test for samples 4032D with admixture of GNP.

Parameter	0.1% Graphene		0.1% Graphene after UV		0.1% Graphene after UV + SOL		0.5% Graphene			0.5% Graphene after UV		0.5% Graphene after UV + SOL	
	Sample_1	Sample_2	Sample_1	Sample_2	Sample_1	Sample_2	Sample_1	Sample_2	Sample_3	Sample_1	Sample_2	Sample_1	Sample_2
Young modulus E [GPa]	1.80	1.79	1.59	1.71	1.74	1.71	1.79	1.71	1.74	1.76	1.76	1.73	1.66
Maximum stress R_m_ [MPa]	59.9	64.0	35.9	32.2	61.7	63	56.9	62.7	57.6	53.6	56.1	62.6	63.6
Strain at maximum load [%]	3.74	3.91	2.23	1.89	3.83	3.90	3.62	4.04	3.76	3.51	3.60	3.87	3.99

**Table 3:** Characteristic parameters obtained in tensile test for samples 4032D with admixture of CNTs.

Parameter	0.1% CNTs		0.1% CNTs after UV		0.1% CNTs after UV + SOL		0.5% CNTs			0.5% CNTs after UV		0.5% CNTs after UV + SOL	
	Sample_1	Sample_2	Sample_1	Sample_2	Sample_1	Sample_2	Sample_1	Sample_2	Sample_3	Sample_1	Sample_2	Sample_1	Sample_2
Young modulus E [GPa]	1.80	1.82	1.63	1.74	1.71	1.72	1.75	1.77	1.74	1.59	1.62	1.61	1.66
Maximum stress R_m_ [MPa]	58.6	56.9	31.2	33.6	62.5	59.8	54.4	54.7	57.3	40.0	34.9	63.3	64.9
Strain at maximum load [%]	3.86	3.62	1.91	2.11	3.92	3.85	3.58	3.47	3.72	2.41	2.16	4.03	4.11

**Table 4:** Values of energies and impact coefficients for samples 3001D, 4032D and 4043D.

Parameter	3001D	3001D after UV	3001D after UV + SOL	4032D	4032D after UV	4032D after UV + SOL	4043D	4043D after UV	4043D after UV + SOL
Energy [J]	4.13	0.50	1.75	5.50	0.50	1.25	4.01	0.50	3.25
Impact strength [J/m^2^]	187.5	22.73	79.56	250.0	22.73	56.82	181.82	22.73	147.73

**Table 5:** Values of energies and impact coefficients for samples 4032D admixed with GNP.

Parameter	4032D +0.1% Graphene	4032D +0.1% Graphene after UV	4032D +0.1% Graphene after UV + SOL	4032D +0.5% Graphene	4032D +0.5% Graphene after UV	4032D +0.5% Graphene after UV + SOL
Energy [J]	6.55	1.38	6.88	8.50	0.75	6.02
Impact strength [J/m^2^]	297.73	62.50	312.50	386.36	34.09	272.73

**Table 6:** Values of energies and impact coefficients for samples 4032D admixed with CNTs.

Parameter	4032D +0.1% CNTs	4032D +0.1% CNTs after UV	4032D +0.1% CNTs after UV + SOL	4032D +0.5% CNTs	4032D +0.5% CNTs after UV	4032D +0.5% CNTs after UV + SOL
Energy [J]	5.88	0.95	2.85	7.10	1.10	4.25
Impact strength [J/m^2^]	267.05	43.18	129.55	322.73	50.01	193.18
